# Case Report: When obstructive sleep apnea is not benign—nasopharyngeal rhabdomyosarcoma in a 3-year-old

**DOI:** 10.3389/fped.2026.1971652

**Published:** 2026-10-26

**Authors:** Tamer Abusido, Mohammed Alghaihab, Atheer Alyami, Abdulmajeed Alfadhel, Rawan Jarwann, Lamia Aldhbiban, Mohammed Alharbi, Alanood Almuhesseny

**Affiliations:** 1 Department of Pediatrics, Ministry of National Guard Health Affairs, King Abdullah Specialized Children Hospital, King Abdulaziz Medical City, Riyadh, Saudi Arabia; 2 King Abdullah International Medical Research Centre, Riyadh, Saudi Arabia; 3 King Saud bin Abdulaziz University for Health Sciences, Riyadh, Saudi Arabia; 4 Department of Pathology, Ministry of National Guard Health Affairs, King Abdullah Specialized Children Hospital, King Abdulaziz Medical City, Riyadh, Saudi Arabia

**Keywords:** case report, diagnostic delay, embryonal rhabdomyosarcoma, epistaxis, nasopharyngeal mass, pediatric obstructive sleep apnea

## Abstract

Obstructive sleep apnea (OSA) is common in children and is most frequently associated with adenotonsillar hypertrophy. However, similar symptoms may occasionally reflect more serious upper airway pathology. We report a previously healthy 3-year-old boy who presented with snoring, mouth breathing, witnessed apneas, and persistent nasal obstruction in the setting of an acute viral upper respiratory illness. Initial flexible nasopharyngeal endoscopy was limited by copious secretions and poor cooperation, with no obvious mass identified. The subsequent development of epistaxis prompted repeat endoscopic evaluation, which revealed a left-sided nasopharyngeal mass. Imaging demonstrated an aggressive parameningeal lesion with skull base erosion and extension into the cavernous sinus. Histopathological examination, supported by positive immunohistochemical staining for desmin, myogenin, and MyoD1, confirmed embryonal rhabdomyosarcoma. The patient received VAC-based chemotherapy and proton radiotherapy, with subsequent tumor regression and stable findings on long-term imaging follow-up; he remained clinically well approximately four years after completion of therapy. This case highlights the potential for diagnostic anchoring when common symptoms of pediatric OSA coexist with apparently benign conditions such as adenotonsillar hypertrophy or an acute respiratory infection. Incomplete nasopharyngeal visualization should not be considered reassuring when symptoms persist or evolve. The emergence of red-flag features, particularly epistaxis, progressive nasal obstruction, or failure to improve as expected, should prompt timely reassessment and consideration of cross-sectional imaging to exclude an underlying structural lesion.

## Introduction

Obstructive sleep apnea (OSA) is a common form of sleep-disordered breathing in children, affecting approximately 2%–5% of the pediatric population (1, 2). Adenotonsillar hypertrophy is the most frequent underlying cause in otherwise healthy children and commonly presents with snoring, mouth breathing, witnessed apneas, and disturbed sleep (1, 2). Although these symptoms usually reflect benign upper airway obstruction, less common structural lesions may produce a similar clinical presentation.

Rhabdomyosarcoma (RMS) is the most common soft tissue sarcoma of childhood, accounting for approximately 4%–5% of pediatric malignancies (3, 4). The head and neck region is a frequent site of involvement, and tumors arising from parameningeal locations, including the nasopharynx, are clinically important because of their proximity to the skull base and potential for intracranial extension (3–5). Early manifestations may be nonspecific and overlap with common pediatric otolaryngologic conditions, including adenoid hypertrophy, rhinitis, and upper respiratory tract infections, potentially delaying recognition of the underlying lesion (4, 6–8)^.^

We report a 3-year-old boy with nasopharyngeal embryonal RMS who initially presented with symptoms suggestive of OSA in the setting of an acute upper respiratory illness. Initial nasopharyngeal endoscopy was limited by secretions and poor cooperation, with the underlying mass identified only after the development of epistaxis prompted repeat evaluation. This case highlights the importance of reassessment when nasopharyngeal visualization is incomplete and emphasizes the diagnostic significance of evolving red-flag features in children presenting with apparently typical symptoms of OSA.

## Case presentation

A previously healthy 3-year-old boy presented to the emergency department with cough and symptoms of an upper respiratory tract infection. Further history from his mother revealed a witnessed episode of breathing cessation during sleep, along with snoring, mouth breathing, and persistent nasal obstruction. These obstructive symptoms had worsened over the preceding two days in association with an acute flu-like illness and reduced oral intake, prompting hospital presentation. Family history was notable for allergic rhinitis in both parents and childhood asthma in the father. His past medical history was otherwise unremarkable. The clinical course from presentation through diagnosis, treatment, and long-term follow-up is summarized in Table 1.

**Table 1:** Clinical course summary.

Time point	Key event
Initial presentation	Snoring, mouth breathing, witnessed apnea, and persistent nasal obstruction, worsening during an acute upper respiratory illness.
Day 0	Initial nasopharyngeal endoscopy was technically limited; no obvious mass identified.
Overnight	Oximetry showed abnormal nocturnal oxygenation.
Day 1	Epistaxis prompted repeat endoscopy, revealing a left nasopharyngeal mass.
Diagnostic work-up	CT/MRI demonstrated an aggressive parameningeal mass with skull-base and cavernous-sinus extension; biopsy confirmed embryonal RMS.
Treatment	VAC-based chemotherapy initiated according to COG D9803; proton radiotherapy planned for local control.
Week 12–39	MRI showed interval regression with residual disease; proton radiotherapy was subsequently delivered, and chemotherapy was completed through week 39.
Long-term follow-up	November 2025 MRI remained stable with no new abnormality; clinically well at the latest oncology follow-up in May 2026.

On examination, he was hemodynamically stable, with normal oxygen saturation in room air and a low-grade fever. Growth parameters were appropriate for age. He was not in respiratory distress; however, persistent mouth breathing and stertor were noted, without stridor. Oropharyngeal examination showed grade 2 tonsillar hypertrophy. A small, mobile left anterior cervical lymph node measuring approximately 1 cm was palpable. Chest examination demonstrated transmitted upper airway sounds without wheeze or crackles. No focal neurological deficit was documented on the initial clinical examination. The remainder of the examination was unremarkable.

Initial laboratory investigations, including complete blood count, coagulation profile, liver and renal function tests, and serum electrolytes, were within normal limits. The patient was admitted for supportive care and rehydration for a presumed viral upper respiratory infection. Both the pediatric pulmonology and otolaryngology teams were involved in his care.

Initial flexible nasopharyngeal endoscopy was limited by copious secretions and poor cooperation, resulting in incomplete visualization of the nasopharynx. No obvious mass was identified, and the initial findings were thought to represent adenotonsillar hypertrophy with a superimposed viral illness. Intranasal corticosteroids and saline irrigation were started, with a plan for reassessment after clinical recovery.

Given limited access to urgent polysomnography, overnight pulse oximetry was performed as an interim assessment. This showed nine desaturation clusters, a desaturation index of 2.6 events/hour, and 9.1% of the recording time with an oxygen saturation below 89%. These findings demonstrated abnormal nocturnal oxygenation and supported the clinical concern for sleep-disordered breathing but did not establish a diagnosis of OSA or identify the anatomical cause of the upper airway obstruction. An early morning blood gas was normal. The following night, the patient developed mild epistaxis, prompting repeat otolaryngologic evaluation. Repeat flexible nasopharyngeal endoscopy revealed a fleshy mass arising from the left side of the nasopharynx, in addition to adenoid hypertrophy. Urgent imaging was obtained for further characterization.

Computed tomography of the head demonstrated an aggressive lesion with bony destruction involving the medial and lateral left pterygoid plates, nasal septum, medial wall of the left maxillary sinus, left lamina papyracea, and sphenoid bone (Figure 1). MRI demonstrated the left nasopharyngeal mass arising from the skull base with extension into the cavernous sinus and associated cervical lymphadenopathy (Figure 2), including a prominent left level II lymph node measuring approximately 1.9 × 1.7 cm.

**Figure 1:**
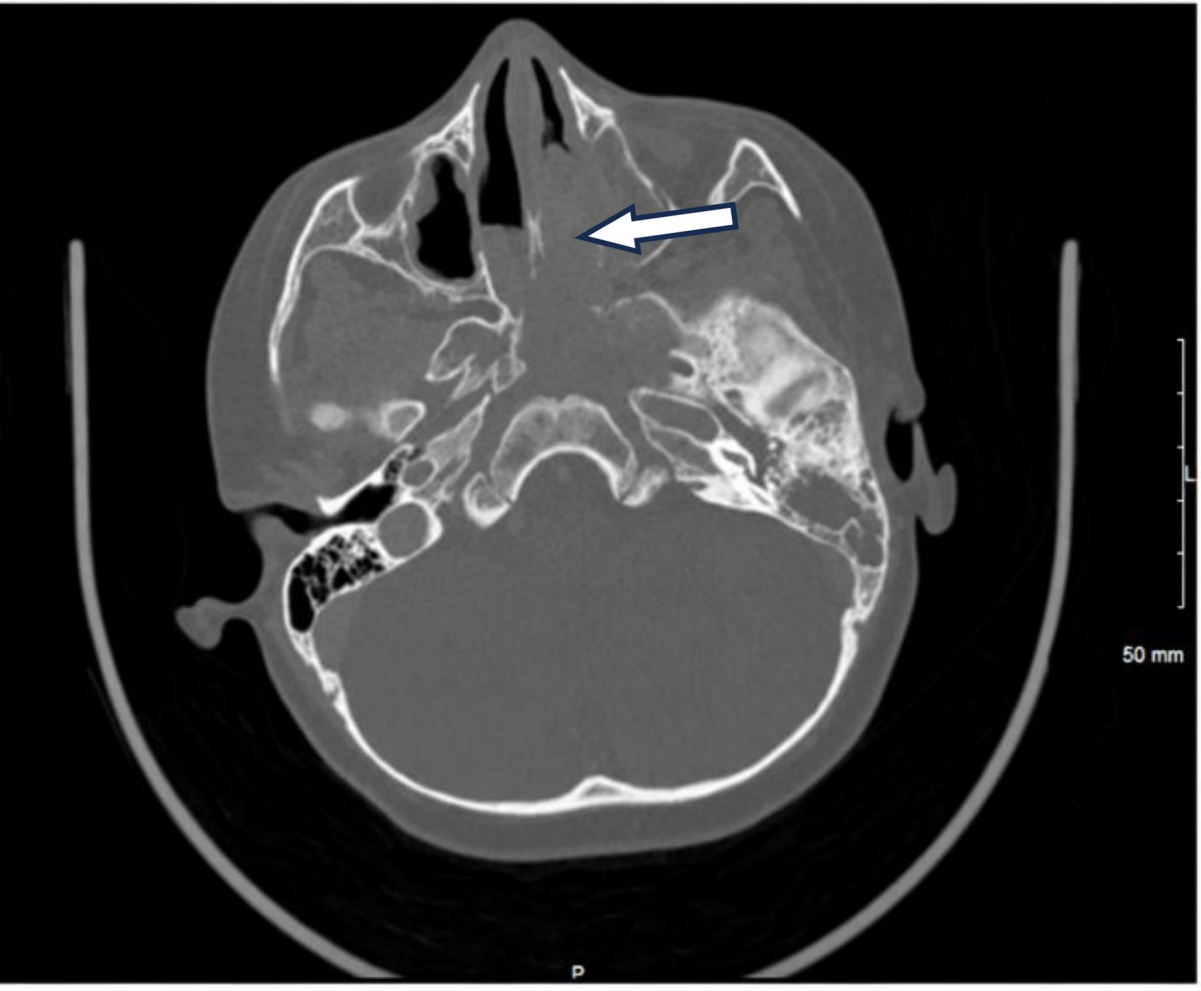
Computed tomography (CT) of the head showing destructive nasopharyngeal lesion. Axial CT image demonstrating the left nasopharyngeal mass (arrow) with associated skull-base involvement.

**Figure 2:**
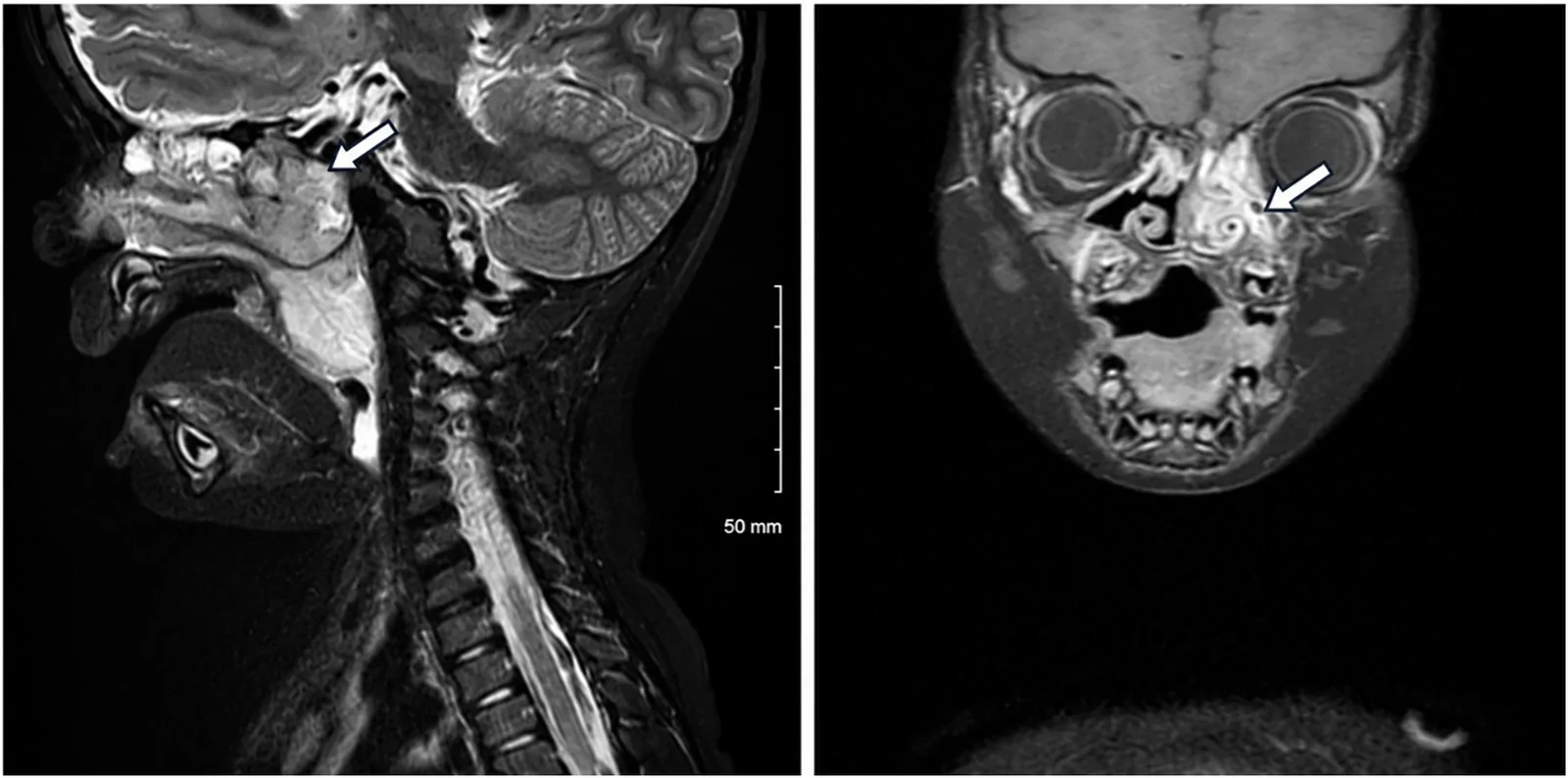
Magnetic resonance imaging (MRI) demonstrating the extent of the nasopharyngeal tumor. Sagittal and coronal MRI images demonstrating the left nasopharyngeal mass with skull-base involvement (white arrows). Extension into the cavernous sinus was identified on the complete MRI examination.

Staging with positron emission tomography/computed tomography showed hypermetabolic activity within the nasopharyngeal lesion and involvement of a left cervical lymph node. Bone marrow aspiration showed no evidence of marrow involvement.

Endoscopic biopsy was performed for definitive diagnosis. Intraoperative frozen section showed a malignant small round blue cell tumor. On permanent sections, the tumor consisted of small round-to-oval cells with hyperchromatic nuclei and scant cytoplasm, with brisk mitotic activity, scattered rhabdomyoblasts, and focal necrosis (Figure 3). Immunohistochemical staining was positive for desmin, myogenin, and MyoD1.

**Figure 3:**
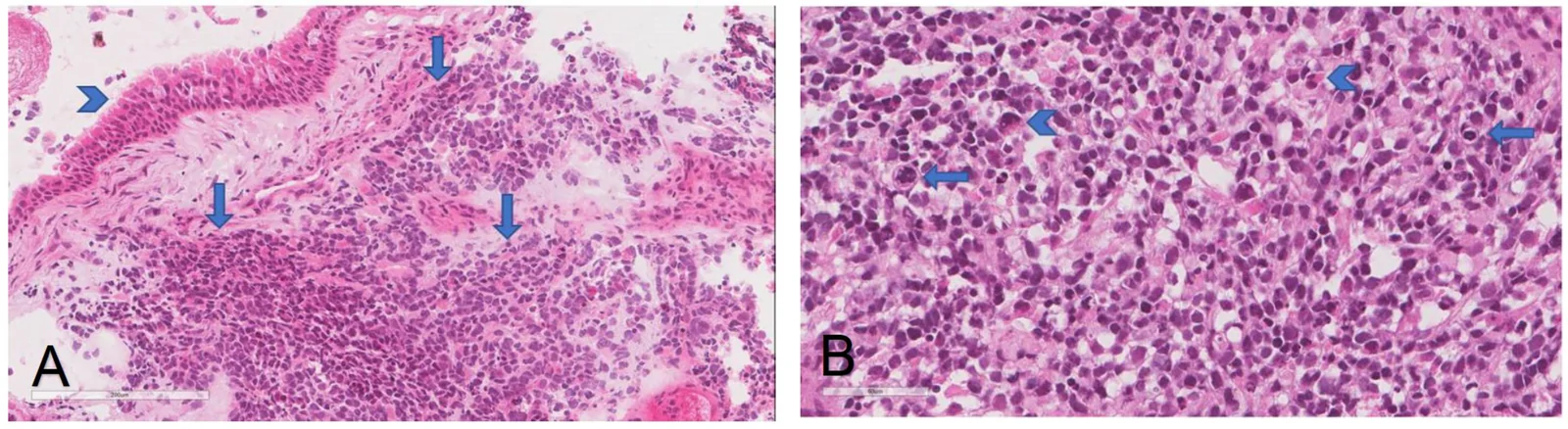
Histopathological section. **(A)** Low-power section showing respiratory-type columnar epithelium (arrowhead) overlying submucosal infiltration by atypical small round tumor cells (arrows). **(B)** High-power hematoxylin and eosin section demonstrating small round-to-oval tumor cells with scattered rhabdomyoblasts (arrowheads) and mitotic figures (arrows).

The final diagnosis was embryonal rhabdomyosarcoma, with no anaplasia identified. Molecular testing using a sarcoma fusion panel detected no gene fusion, including no FOXO1 fusion.

The patient was classified as having intermediate-risk, Stage III, Group III parameningeal rhabdomyosarcoma and was started on chemotherapy according to the Children's Oncology Group (COG) D9803 protocol, using a VAC-based regimen comprising vincristine, dactinomycin, and cyclophosphamide, administered in three-week cycles. Local control with radiotherapy was planned following the initial chemotherapy cycles, with reassessment at week 12. Proton therapy was preferred because of the patient's young age and the head and neck location of the tumor, with the aim of limiting radiation exposure to surrounding structures and reducing potential long-term treatment-related morbidity. At week 12, follow-up MRI demonstrated interval regression of the left nasopharyngeal mass with residual disease. The patient subsequently travelled to the United States for proton radiotherapy; however, international travel and visa-related delays affected the planned treatment schedule. The available institutional records did not provide sufficient information to retrospectively establish the rationale for the timing of radiotherapy in relation to the COG D9803 protocol recommendations for parameningeal disease with intracranial extension. Details of the proton radiotherapy dose, fractionation, and target volumes were not available in our institutional records. He later returned to our institution and completed the planned chemotherapy course through week 39.

During treatment, he experienced febrile neutropenia complicated by Klebsiella bacteremia, from which he recovered with antimicrobial and supportive therapy. During subsequent follow-up, serial imaging demonstrated continued reduction and later stability of the treated lesion. The most recent available face and neck MRI, performed in November 2025, showed stable findings with no new abnormality. At his latest oncology follow-up in May 2026, approximately four years after completion of therapy, he remained clinically well with no new concerns and no documented neurological or cranial nerve abnormalities. No recurrence or new disease was documented during this follow-up period.

## Discussion

This case illustrates an uncommon cause of symptoms that initially appeared typical of pediatric OSA. Snoring, mouth breathing, witnessed apneas, and nasal obstruction in a 3-year-old would usually raise adenotonsillar hypertrophy high on the differential diagnosis (1, 2). In our patient, the presence of adenotonsillar hypertrophy together with an acute upper respiratory infection provided a reasonable initial explanation for his symptoms. However, this also created the potential for diagnostic anchoring when the initial nasopharyngeal examination was incomplete.

RMS is the most common soft tissue sarcoma in children, and the head and neck region is one of its recognized sites of involvement (3, 4). Tumors arising from parameningeal sites, including the nasopharynx, are particularly important because of their proximity to the skull base and potential for intracranial extension (3–5). Their early presentation may be nonspecific, with symptoms overlapping with common pediatric otolaryngologic conditions such as adenoid hypertrophy, rhinitis, and upper respiratory tract infections (4, 6–8). Nasopharyngeal RMS presenting with symptoms of OSA has been reported previously (7, 8), making the diagnostic pathway in the present case particularly relevant.

The histopathological subtype in our patient was embryonal RMS. According to the fifth edition of the World Health Organization (WHO) Classification of Soft Tissue and Bone Tumours, RMS is classified into embryonal, alveolar, pleomorphic, and spindle cell/sclerosing types (10). In our patient, microscopy demonstrated a small round cell tumor with rhabdomyoblastic differentiation, while immunohistochemistry showed positivity for desmin, myogenin, and MyoD1. No anaplasia was identified. Molecular testing using a sarcoma fusion panel detected no gene fusion, including no FOXO1 fusion. Together, the morphological, immunohistochemical, and molecular findings supported the diagnosis of embryonal RMS. The absence of a FOXO1 fusion is consistent with the molecular profile commonly encountered in embryonal RMS; however, fusion-negative RMS represents a genetically heterogeneous group. Large genomic studies have identified recurrent alterations involving the RAS pathway and other genes, including TP53 and MYOD1, in subsets of fusion-negative tumors, with some alterations carrying potential prognostic significance (11). Additional molecular alterations have also been described across rhabdomyosarcoma subtypes (12). These additional alterations were not specifically evaluated in our patient, and the negative sarcoma fusion panel should therefore not be interpreted as excluding other genomic abnormalities.

An important feature of this case was that the tumor was not identified during the initial flexible nasopharyngeal endoscopy. Visualization was limited by copious secretions and poor cooperation, both of which are common practical challenges when examining young children. The absence of an obvious mass during an incomplete examination should therefore not be considered equivalent to adequate visualization of the nasopharynx. In our patient, the subsequent development of epistaxis prompted repeat endoscopy and ultimately led to identification of the lesion.

Epistaxis was the main clinical turning point. Persistent or progressive nasal obstruction, unexplained epistaxis, cervical lymphadenopathy, failure to improve as expected, or an incomplete nasopharyngeal examination should prompt reconsideration of the initial diagnosis (6–8)^.^ When these features are present, repeat endoscopic assessment and cross-sectional imaging should be considered rather than attributing persistent symptoms solely to adenotonsillar hypertrophy or an intercurrent respiratory infection.

Imaging was essential once the mass was identified. CT demonstrated extensive bony destruction, while MRI better defined the skull base involvement and extension into the cavernous sinus. These findings established the extent of the lesion and were consistent with an aggressive parameningeal tumor (5, 9). Nevertheless, imaging alone cannot establish the histological diagnosis, and tissue biopsy remains essential.

Once a nasopharyngeal mass is identified in a child, the differential diagnosis is broad and includes both benign and malignant lesions. Benign considerations include prominent adenoidal tissue, antrochoanal polyps, juvenile nasopharyngeal angiofibroma, and nasopharyngeal cysts such as a Thornwaldt cyst. Malignant considerations include rhabdomyosarcoma, lymphoma, nasopharyngeal carcinoma, and other pediatric small round cell tumors such as Ewing sarcoma/PNET and neuroblastoma. Age, clinical presentation, imaging characteristics, and the presence of local invasion can help narrow the differential, but tissue diagnosis remains essential when malignancy is suspected.

Overnight pulse oximetry in this patient demonstrated abnormal nocturnal oxygenation and was obtained because urgent polysomnography was not readily available. Current pediatric OSA guidance recognizes overnight oximetry as a useful adjunct to clinical assessment when access to polysomnography is limited, but it should not be used in isolation to diagnose or exclude OSA (2). In our patient, the abnormal oximetry findings supported the clinical concern for sleep-disordered breathing but did not establish the diagnosis of OSA or identify the anatomical cause of the upper airway obstruction. Sleep-related investigations should therefore be interpreted alongside the clinical and upper airway assessment, particularly when symptoms persist or atypical features emerge.

Nasopharyngeal rhabdomyosarcoma presenting with snoring or symptoms suggestive of OSA has been reported previously. Love and MacKay described a child with rhabdomyosarcoma presenting with symptoms of sleep-disordered breathing, while Adeel et al. similarly reported rhabdomyosarcoma presenting with features initially suggestive of obstructive sleep apnea (7, 8). The present case adds a different diagnostic perspective. Here, a typical OSA phenotype occurred alongside adenotonsillar hypertrophy and an acute respiratory illness, providing a plausible benign explanation, while the initial nasopharyngeal examination was technically incomplete because of secretions and poor cooperation. Abnormal overnight oximetry supported the presence of sleep-disordered breathing but did not identify its anatomical cause. The subsequent development of epistaxis changed the clinical picture and prompted repeat endoscopy, revealing the nasopharyngeal mass. This sequence illustrates how diagnostic anchoring on a common explanation, particularly when the initial examination is incomplete, may delay recognition of an uncommon structural lesion.

This report has several limitations. As a single case, its findings are primarily illustrative. The initial nasopharyngeal examination was technically incomplete, and polysomnography was not performed. Detailed proton radiotherapy parameters were unavailable because treatment was delivered at an external center, and long-term follow-up records did not specifically document the resolution of the presenting sleep-related symptoms.

## Parent perspective

The patient's family expressed their gratitude for the multidisciplinary care provided throughout his diagnosis and treatment. They particularly appreciated the teamwork and coordination between the local treating team and the specialized center in the United States, which facilitated continuity of care across the different stages of his treatment.

## Conclusion

In children with presumed OSA, incomplete nasopharyngeal visualization should not be considered reassuring when symptoms persist or evolve. New red-flag features, particularly epistaxis or progressive nasal obstruction, should prompt repeat upper airway evaluation and consideration of cross-sectional imaging to exclude an underlying structural lesion.

## Data Availability

The datasets presented in this article are not readily available because of ethical and privacy restrictions. Requests to access the datasets should be directed to the corresponding author.
